# Analysis of gene expression in cotton fiber initials

**DOI:** 10.1186/1471-2229-7-22

**Published:** 2007-05-16

**Authors:** Earl W Taliercio, Deborah Boykin

**Affiliations:** 1 USDA/ARS, 3127 Ligon St, Raleigh, NC, 27607, USA; 2 USDA/ARS, 141 Experiment Station Rd., Stoneville, MS 38776, USA

## Abstract

**Background:**

Cotton (*Gossypium hirsutum *L.) fibers are trichomes that initiate from the ovule epidermis. Little is known about the developmental pathway causing fiber to differentiate from ovular epidermal cells even though limits on the number of cells that differentiate into fiber will limit yield.

**Results:**

A method was developed to isolate RNA from fiber initials 1 day post anthesis (dpa). Complementary DNA libraries representing 1 dpa fibers and other cotton tissues were sequenced and analyzed. Assembly of *G. hirsutum *Expressed Sequenced Tags (ESTs) identified over 11,000 sequences not previously represented in GenBank. New genes identified among these ESTs were represented on microarrays. The microarrays were used to identify genes enriched in fiber initials (1 dpa fibers) and elongating fibers. Analyses of Gene Ontologies (GO) of differentially expressed genes determined that terms associated with the "membranes" were statistically over represented among genes increased in expression in fiber initials and 10 dpa fibers. Staining ovules with a fluorescent dye confirmed an increase in Endoplasmic Reticulum (ER) occurred in fiber initials on the day of anthesis, persisted through 3 dpa and was absent in a *fiberless *mutant. Two genes similar to the CAPRICE/TRIPTYCHON (CPC) gene that inhibits differentiation of leaf trichomes in *Arabidopsis *were also characterized. Genes associated with novel regulation of brassinosterols, GTP mediated signal transduction and cell cycle control and components of a Ca^+2 ^mediated signaling pathway were identified. Staining of cellular Ca^+2 ^indicated that fiber initials had more Ca^+2 ^than other ovule cells supporting a role for Ca^+2 ^in fiber development.

**Conclusion:**

Analysis of genes expressed in fiber initials identified a unique stage in fiber development characterized by an increase in ER and Ca^+2 ^levels that occurred between 0 and 1 dpa. The gene similar to CPC has a MYB domain but appears to lack a transcription activating domain similar to the *Arabisopsis *gene. The method used to stain the ER also can be used to count fiber initials and showed fiber cells develop from adjacent cells unlike leaf trichomes.

## Background

Trichomes initiated from cotton ovule epidermal cells develop into spinnable fiber. Little is known about fiber initiation even though the number of ovule epidermal cells that differentiate into fiber impacts yield of this important crop. Cotton fibers are single cells that differentiate nearly synchronously through 4 over-lapping stages of development [1]. From about -2 dpa (days post anthesis) to 2 dpa ovular epidermal cells differentiate into fiber initials, from 2 dpa to 21 dpa fibers rapidly elongate up to 5 cm in length, beginning about 16 dpa massive amounts of cellulose are deposited in the secondary cell wall and finally the fiber matures and dries.

Fiber initials represent a minority of epidermal ovule cells and identification of epidermal cells that develop into fibers is difficult before 0 dpa. Ovules cultured *in-vitro *become competent to produce fiber in response to auxin and giberellic acid 2 days before anthesis [1]. Fiber initiation also requires brassinosterol production [2]. Fiber differentiation is evident *in-vivo *by -1 dpa when microtubules reorient in epidermal cells destined to differentiate into fibers [3]. On the day of anthesis the amount of golgi bodies and ER increase [3,4]. By 1 dpa, fiber initials bulge from the surface of the ovule. Protein biosynthesis and nucleoli size increase in very young fibers [5]. *In-vitro *cultured ovules indicated that mRNA synthesis is required for fiber initiation up to 2 dpa and the ovules remained competent to initiate fibers up to 5 dpa [6-9]. Conservatively, the period of fiber initiation ends at 2 dpa and may extend to 5 dpa. Fiber initiation requires transcription and therefore transcription factors are likely to play an important role in fiber initiation. The Myb109 and MYB2 transcription factors are expressed in fiber initials [10]. The Myb2 transcription factor is able to complement *Arabidopsis thaliana *trichome mutants and activate expression of R22-like (RDL) gene expressed in fiber initials [11,12]. Additionally, the RDL gene along with genes involved in cell structure, long chain fatty acid biosynthesis and sterol biosynthesis have been identified that are absent or reduced in a fiberless mutant of cotton [13]. Most of these genes are expressed in 1 dpa ovules. Evaluation of fiberless cotton mutants has identified genes differentially expressed in very young fiber, including transcription factors shown to play roles in fiber development [14,15]. A second rounds of fiber initiation occurs that produces the short linters or fuzz fibers.

Fiber elongation occurs by a diffuse growth mechanism [16]. Many genes expressed during the elongation stage of fiber differentiation relate to cell expansion, cell wall loosening, and osmoregulation [17-20]. Ovule culture studies confirmed a role for brassinosterols during fiber elongation in addition to fiber initiation [21]. Genomic analyses by Shi *et al*. indicated that ethylene plays an important role during fiber elongation [22]. The role for ethylene in fiber elongation was confirmed when longer fibers were obtained with the addition of ethylene to ovule culture. An increase in cellulose and expression of genes encoding cellulose synthase marks the end of the rapid elongation stage of the fiber development.

In this investigation a method to isolate RNA from 1 dpa fiber initials is presented. Genes expressed during fiber initiation and elongation were identified using a custom DNA microarrays representing over 11,000 genes, many of which were originally identified for this study. Genes with known patterns of expression were used to validate the microarray data. Additionally, the differential expression of selected genes was also confirmed by RNA blot analysis and semiquantitative PCR. Analyses of gene ontologies (GO) indicated that endomembranes and a GTP signaling pathway increased in developing fibers. Other genes not falling into the GO categories that are differentially regulated during fiber development also provided insight into fiber initiation and elongation. Genes associated with Ca^+2 ^signaling pathways are differentially regulated during fiber initiation and elongation. Differentially regulated genes similar to GLABRA2 (GL2) and Caprice (CPC) which play a role in *Arabidopsis *trichome and root hair development were also identified [23-25]. These results were supported by histological methods and more detailed analysis of expression of selected genes to broaden our understanding of cotton fiber development.

## Results

### Isolation of RNA from 1 dpa fiber

**Figure 1:**
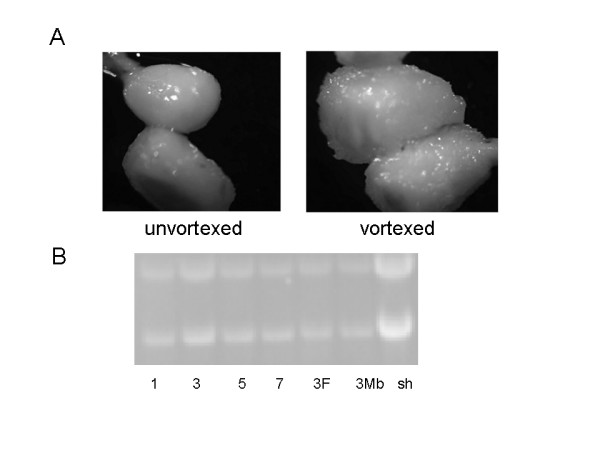
RNA isolated from frozen ovules. One sample was mixed with glass beads and vortexed and a picture was taken. A picture was taken of the other frozen ovules without addition of glass beads or vortexing (A). Polyribosomal RNA was isolated from vortexed 1 dpa, 3 dpa, 5 dpa or 7 dpa ovules and shoots. Free-polyribosomal (F) or membrane bound polyribosomal (Mb) RNA was isolated from vortexed 3 dpa ovules. The RNA was separated on a 1.2% gel and visualized by staining with ethidium-bromide.

**Figure 2:**
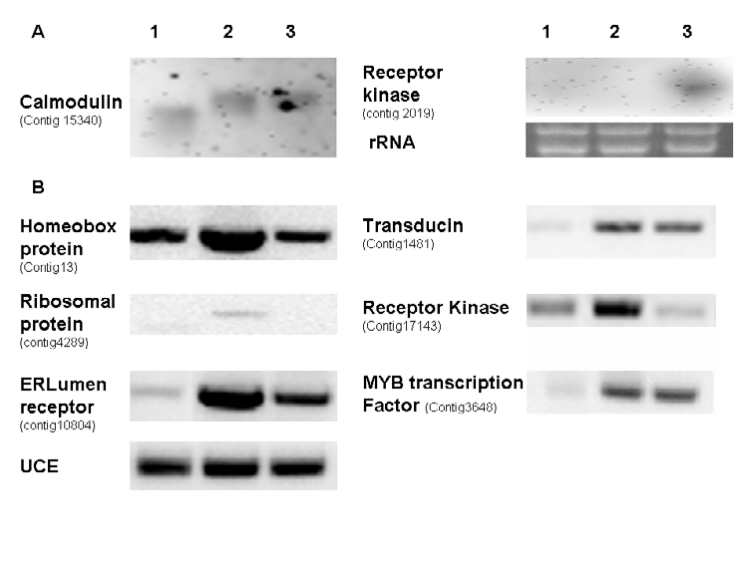
Validation of expression of selected genes. Lanes 1, 2 and 3 represent RNA from 0 dpa ovules, 1 dpa fibers and 10 dpa fiber, respectively. Panel A shows RNA blot analysis of 2 ug of total RNA hybridized as indicated. Ethidium bromide stained rRNA bands of the RNA used in these experiments are also shown. Panel B Shows semiquantitative PCR of the indicated transcript. UCE was used a loading control.

One goal of these experiments was to extend the cotton dbEST to represent a range of tissues including fiber initials and identify genes important to fiber initiation. ESTs representing whole ovules have recently been analyzed [26]. Isolating RNA relevant to fiber initiation was difficult because only about 25% of cells on the ovule epidermis differentiate into fibers. While whole ovule RNA included RNA from fiber initials, the fiber initial RNA was substantially diluted by RNA from other ovular cell types. A method was developed to isolate RNA from 1 dpa fibers based on a protocol used to isolate RNA from root hairs in *Medicago *[27]. Ovules (0–7 dpa) were frozen in an excess of liquid nitrogen, glass beads were added and the mixture vortexed for 5 min. Fig. 1A shows that the frozen and vortexed ovules remain substantially intact. RNA could be isolated from the vortexed 1 dpa ovules and was similar in quality to RNA from shoots based on the easily visualized rRNA bands (Fig. 1B). RNA could not be isolated from vortexed 0 dpa ovules; indicating that fiber initials had to protrude above the ovule surface for the method to work. RNA could not be isolated from 1 dpa fiberless ovules indicating that RNA is derived from fiber initials. Semiquantitative rt-PCR amplification of genes differentially expressed in 1 dpa shown in Fig. 2B (primers in Table 1) and microarray analysis of genes known to be expressed in fiber initials confirm that this RNA was enriched in fiber initial transcripts. Complementary DNA libraries prepared from RNA representing 1 dpa fiber were sequenced. Complementary DNA libraries representing roots and stems of various ages were also sequences. Libraries were also sequenced that represented normalized cDNA populations derived from pooled RNAs of multiple tissues including 1 dpa fiber. All of the tissues represented by these ESTs (including other unnormalized libraries) are shown in Table 2. Over 66,000 new cotton EST sequences were deposited in GenBank and the accession numbers are shown in Table 2.

**Table 1:** List of primers.

Gene	forward primer (5'-3')	reverse primer (5'-3')
Contig13	CGGTCGATATTGTTGCAATG	AGGAGAAAGGCAGCAGCTAA
Contig4289	GATGTCGAGGAGAACATTTGC	TTGGTGCCAACAAAAATCAA
Contig10804	TGGTACATGCGGTATCACAAA	TCAAAGCACATTGACCACCT
UCE	CCATTCAAAGGCCTCCCCAAGGTTT	CCACACCACCACTTTATCAAAGGATCCAA
Contig1481	ACGATCAGGGTGTGGAGAAG	GTGAGATGACCCCCTGAAAA
Contig17143	AAACCCCCAAAATGGCTAAC	TCACAGTGCCATAGAGATGGA
Contig6348	CTCGTCTCCTTCAAGGTTGG	TCCCAAACTTTTTCAGATTCC

The primers used to amplify selected transcripts.

**Table 2:** Summary of cDNA libraries.

Description	no. EST	unique contigs	singletons	cultivar	Accession numbers
fiber 5 dpa (FFT)	274	16	59	DES119	DW223534–DW223807
lower stem (between the root and first leaf node 7 weeks after planting)	1029	12	163	DES119	DW223808–DW224836
lower stem (between the root and first leaf node 3 weeks after planting)	3203	22	287	DES119	DW224837–DW228039
fiber initials 1 dpa (from membrane bound polyribosomes)	1641	24	71	DES119	DW228040–DW229680
0 dpa ovules from the fiberless mutant (SL1-7-1)	1727	37	156	SL1-7-1	DW229681–DW231407
0 dpa ovules from DES119	1749	0	148	DES119	DW231414–DW233162
root 3 week after planting (from free polyribosomes)	2081	14	191	DES119	DW233163–DW235243
root 3 week after planting (from membrance bound polyribosomes)	669	29	82	DES119	DW235244–DW235912
Roots 10–12 weeks after planting	2818	27	222	DES119	DW235913–DW238730
stems 7 weeks after planting (membrane bound polyribosomes)	519	5	4	DES119	DW238731–DW239249
stems 3 week after planting	3087	0	282	DES119	DW239250–DW242336
stems 3 week after planting (from membrane bound polyribosomes)	690	0	88	DES119	DW242337–DW243026
stems 3 weeks after planting (unsuccesful normalization)	1036	0	63	DES119	DW243027–DW244062
Young fiber (1–5 dpa)	1583	0	10	DES119	DW244063–DW245645
random primed normalized	19600	704	3325	DES119	DW476068–DW495667
oligo dT primed normalized	25015	675	3450	DES119	DW495668–DW520682

Description of cDNA libraries, the number of ESTs sequences in each library, unique contigs composed exclusively of ESTs from library, singletons unique to library, cotton cultivar used to make cDNA, GenBank accession numbers of ESTs sequences.

### Analysis of ESTs

Assemblies of *G. hirsutum *ESTs identified 4,303 contigs and 8,601 singletons not previously represented among *G. hirsutum *EST in GenBank (dbEST). About 43% of the contigs and singletons in this assembly were derived solely from ESTs sequenced for this study. Details of the number of new contigs and singletons identified among these ESTs are shown in Table 2. The high rate of recovery of new genes indicated the importance of representing multiple tissues with normalized libraries. Contigs composed of ESTs solely from these new libraries should be enriched in genes represented in 1 dpa fiber and other tissues represented in the EST libraries. Approximately 11,000 sequences, most unique to this assembly, were represented on a microarray in triplicate or quadruplicate.

### Validation of microarray data

Expression profiling was performed using the cotton microarray to assess changes in gene expression in fiber initials compared to whole ovule and elongating fiber. Fluorescently labeled cDNA prepared from 1 dpa fiber, representing the period of fiber initiation, was hybridized against 6 microarrays. Three of these arrays were also hybridized with the complementary labeled cDNA derived from 1 dpa ovule RNA and the other three with the complementary labeled cDNA derived from 10 dpa fiber RNA. Benchmark genes, many of which are known to be differentially regulated during fiber initiation and elongation, were represented on the microarray to validate expression (additional file 1) [13]. An mRNA encoding an Acetyltransferase (GhACY) and a FIDDLEHEAD homolog (GhFDH) were more abundant in 1 dpa fiber compared to 1 dpa ovules consistent with reported expression of these genes. RNA encoding a serine carboxypeptidase (GhSCP) and a Beta-tubulin (Ghtub) were increased in 10 dpa fiber compared to 1 dpa fiber. Expression of a GhSCP and Ghtub were consistent with genes increased in expression in 5 dpa fiber as previously reported. Similarly, mRNAs encoding a cellulose synthase, sterol-C-methyltransferase, flavanone 3-beta-hydroxylase, heat shock protein 70 and another serine protease-like protein were not increased in 1 dpa fiber. Nor were these mRNAs increased in 10 dpa fiber with the exception of the mRNA encoding the serine protease-like protein. These data are in good agreement with the previously reports that showed no differential expression of these genes in 5 dpa fiber.

Expression of genes encoding the transcription factors MYB109, MYB(2–6) comported well with published data (additional file 1) [10,11]. Messenger RNA encoding MYB109 and MYB2 increased in 1 dpa fiber compared to 1 dpa ovules and persisted in 10 dpa fibers. RNA encoding a RD22-like protein (GhRDL) fell slightly below the threshold for increase at 1 dpa but was substantially increased in 10 dpa fibers. The GhRDL gene had been shown to be activated by the MYB2 homolog in *Gossypium arboreum*. These genes were included on the microarray because their expression has been investigated in young fiber. The agreement of the known expression of these genes with expression of these genes on microarrays validated the microarray results.

### Global analysis of gene expression

Genes that vary greater than two fold in expression between 1 dpa fiber and ovules or 1 dpa fiber and 10 dpa fiber are included in additional file 1. Also included in additional file 1 is the significance of the variation. Comparison of expression of genes between 1 dpa fiber and 1 dpa ovules identified 248 transcripts that were down regulated in 1 dpa fiber and 376 transcripts that were up regulated in 1 dpa fiber. Comparison of expression of genes between 1 dpa fiber and 10 dpa fiber identified 390 transcripts that were down regulated in 1 dpa fiber and 165 transcripts that were up regulated in 1 dpa fiber. There were 59 transcripts that were upregulated in 1 dpa fiber compared to both 1 dpa ovules and 10 dpa fiber.

One advantage of using microarrays to profile gene expression was the opportunity to evaluate the expression of large numbers of genes. However, it can be difficult to comprehend the simultaneous change in expression of so many genes. The use of GO provides a tool to grasp the meaning of the changes of expression of large sets of genes [28]. Gene ontologies are definitions of genes using a well defined species-independent vocabulary. GO were not available for most of the genes represented on the microarray since selected genes were not in GenBank. GO are available for many *Arabidopsis *genes so *Arabidopsis *cognates of the genes represented on the microarray were used to analyze the ontologies of genes differentially expressed in fiber initials and elongating fibers. The GO of *Arabidopsis *cognates were analyzed using GOstat [29]. GO from genes increased at least two fold in relevant tissues were compared with all other GO from genes on the microarray. The statistical analysis identifying terms more or less prevalent in the GO representing genes up-regulated in 1 dpa or 10 dpa fiber is shown in Table 3 and 4, respectively. Pathways associated with biosynthesis and particularly protein biosynthesis were enriched in 1 dpa fiber. These results agreed with published data. The rapidly differentiating and growing young fibers are sites of active protein synthesis [3]. Young fibers also have large nucleoli to support rapid protein synthesis [5]. An increase in nonmembrane bound organelles and factors related to biogenesis and membranes were also reported.

**Table 3:** GO analysis of fiber initials.

**Description**	**GO**	**Count (374)**	**Total (5494)**	**P-Value increased**	**P-Value decreased**
ribosomes	GO:0005840	31	82	2.77E-25	
structural constituents of ribosome	GO:0003735	31	83	3.98E-25	
ribonuceoprotein complex	GO:0030529	31	92	1.21E-21	
protein biosynthesis	GO:0006412	44	167	2.33E-21	
macromolecule biosynthesis	GO:0009059	48	223	2.75E-16	
intracellular nonmembrane-bound organelle	GO:0043232	34	140	4.19E-14	
biosynthesis	GO:0009058	65	427	1.33E-10	
cytosol	GO:0005829	20	50	1.73E-09	
establishment protein localization	GO:0045184	22	93	2.15E-08	
protein transport	GO:0015031	21	87	2.61E-08	
cellular biosynthesis	GO:0044249	54	364	3.76E-08	
cell organization and biosynthesis	GO:0016043	36	208	1.19E-07	
localization	GO:0051179	67	515	3.72E-07	
intrinsic to membrane	GO:0031224	31	174	5.36E-07	
transport	GO:0006810	66	510	5.83E-07	
cytoplasmic organization and biogenesis	GO:0007028	11	25	8.83E-06	
DNA Binding	GO:0003677	6	486		1.90E-05
carrier activity	GO:0005386	25	152	0.000107	
intracellular transport	GO:0046907	21	118	0.000108	
establishment of cellular localization	GO:0051649	21	118	0.000108	

Analysis of ontologies of genes increased in expression in 1 dpa fiber initials compared to intact ovules. A description of the GO category, numbers of genes (count) differentially regulated, total number of genes with indicated GO and confidence in increase or decrease are shown.

**Table 4:** GO analysis of 10 dpa fiber.

**description**	**GO**	**Count (313)**	**Total (4351)**	**P-Value increased**	**P-Value decreased**
organelle	GO:0043226	66	1691		7.30E-09
intracellular organization	GO:0043229	66	1691		7.30E-09
intracellular	GO:0005622	78	1869		7.30E-09
Membrane bound organelles	GO:0043227	63	1635		7.30E-09
hydrolase activity	GO:0016798	18	72	1.61E-06	
cytoplasm	GO:0005737	62	1504		1.63E-06
plastid	GO:0009536	21	784		8.22E-06
hydrolyzing-O glycosyl bonds	GO:0004553	16	68	1.09E-03	
endomembrane system	GO:0012505	70	616	1.31E-03	
cellular metabolism	GO:0044237	69	1437		1.31E-03
membrane	GO:0016020	98	947	1.50E-03	
cell wall modification	GO:0009831	3	4	4.41E-02	

Analysis of ontologies of genes increased in expression in 10 dpa fiber initials compared to 1 dpa fiber. A description of the GO category, numbers of genes (count) differentially regulated, total number of genes with indicated GO and confidence in increases or decreases are shown.

GO analysis of genes upregulated in 10 dpa fiber indicated that transcripts associated with organelles (excluding cell membrane and nucleus) decreased (Table 4). An increase was observed for genes associated with cell wall modifications as would be expected for these rapidly growing cells. Even with a decrease in organelles, there was an increase in membranes, consistent with the need for rapidly expanding cell membrane.

Another excellent source of GO annotated genes was UniProt [30,31]. UniProt cognates of the genes represented on the microarray were also used to analyze the ontologies of genes differentially expressed in fiber initials and elongating fibers. The UniProt cognates were analyzed using GOstat [29]. The GO analyses of the UniProt cognates were similar to the analyses with the *Arabidopsis *cognates with one exception. Six genes (Contig10324, Contig85, Contig16430, Contig2338, Contig6782, Contig1658) involved in a small GTPase mediated signal transduction pathway (P = 0.0169) were up-regulated in 1 dpa fiber and persisted in 10 dpa fiber [32,33]. These genes may also play roles in vesicle trafficking.

### Validation of GO analysis

**Figure 3:**
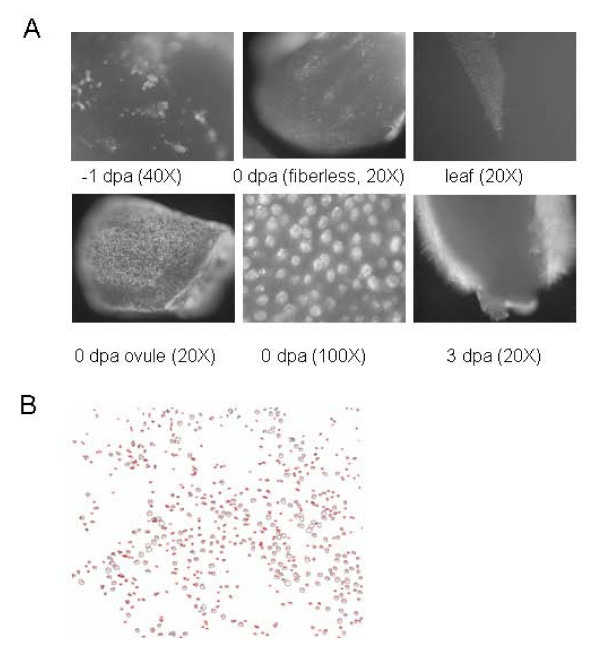
Ovules and leaves were stained with DiOC. Panel A. The wild type ST4793R was used unless otherwise noted. Water stained controls showed no auto fluorescence (data not shown). The fiberless mutant SL1-7-1 was used where indicated. The magnification is indicated in parentheses under the picture. Panel B. An image of a 20× magnification was digitalized and stained features identified, highlighted in red and counted using ImageJ.

The GO analyses identified a consistent increase in membrane associated components (designated "intrinsic to membrane", "endomembrane" and "membrane") indicating that membrane increase played a role in early fiber development consistent with the increased ER reported in fiber initial EM studies [4]. To test this hypothesis that ER increased during fiber initiation, the ER from -1 dpa and 0 dpa ovules was stained with 3, 3'-Dihexyloxacarbocyanin iodide (DiOC) [34]. Unstained ovules gave little or no autofluorescence (data not shown). No easily distinguishable staining patterns were observed on -1 dpa ovules, but by 0 dpa a distinct subset of ovule epidermal cells were stained with DiOC (Fig. 3A). Higher magnifications of stained 0 dpa ovules clearly showed that the small fibers were preferentially stained compared to other epidermal cells (Fig. 3A). A view of the cut section of a stained 3 dpa ovule illustrated the contrast between the small fibers and other ovule cells and that the increase in ER persisted in fibers to at least 3 dpa (Fig. 3A). DiOC also stains mitochondria but the staining pattern of fiber initials indicates most of the staining was ER.

The ER of 0 dpa ovules from fiberless mutant was also stained. No coherent staining was observed in the fiberless mutant (Fig. 3A). The collective evidence supported a rapid increase in ER between -1 and 0 dpa in nascent fibers, which was absent in the fiberless mutant.

The ER of expanding leaves was also stained with DiOC (Fig. 3A). Unstained controls showed minimal autofluorescence that was easily distinguishable from the fluorescence due to DiOC staining (data not shown). Leaf trichomes were not stained more intensely than the surrounding cells, indicating they were not enriched in ER relative to nearby cells. These images required very long exposures (~26 s) compared to the DiOC stained ovules (50 ms).

There is currently no easy way to directly measure fiber density. An image of a 0 dpa DiOC stained ovule was digitalized and the stained cells identified and counted (Fig. 3B). DiOC staining provided sufficient contrast to identify and count fiber initials. We are currently developing high through-put methods to count fiber initials in defined areas.

### Transcription factor and other genes differentially expressed in fiber initials

Many genes were not included in the GO analysis though they were clearly differentially expressed. Pathways associated with these may have not been identified because of poor representation of the pathway on the microarray of lack of annotated genes associated with these pathways, among many possible reasons. Fifty nine genes were identified that were up-regulated in the fiber initials, and were less abundant in whole ovule and down-regulated in 10 dpa fiber. Four genes that gave insight into various aspects of fiber initiation were identified by eliminating genes with poor annotations and genes with functions already shown to be important in fiber initiation (Contig3145, Contig1481, Contig3407, and Contig7833). A prohibitin that potentially regulates the cell cycle was identified [42]. A MATE efflux protein that may play a role in lateral root initiation [36] was identified. Semiquantitative rt-PCR confirmed that a transducin and a ribosomal protein were expressed at the highest levels in 1 dpa fiber compared to whole ovules and 10 dpa fiber (Fig. 2B). Transducins are a component of a GTP-mediated signaling pathway not identified in the GO analysis [32,33]. A putative steroid sulfotransferase mRNA was also upregulated in fiber initials [37].

**Figure 4:**
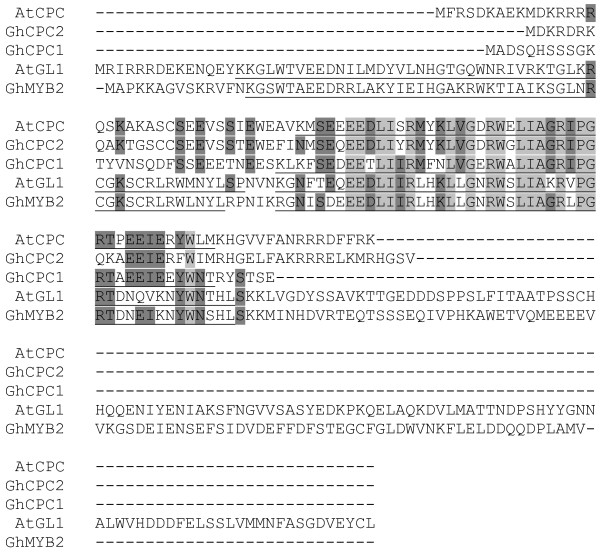
Alignment of CPC and other MYBs: GhCPC1(contig16590), GhCPC2(Contig17149), AtCPC(NP_182164), AtGL1(NP_189430), GhMYB2(translation of MYB2). Regions of identity shared by all clones lightly shaded. Regions shared by majority of clones darkly shaded. MYB motifs underlined.

Many of the benchmark genes used to validate expression of the microarray were MYB type transcription factors. The MYB transcription factors (MYB2 and MYB109) that play a role in fiber initiation were up-regulated in 1 dpa fiber relative to whole ovules and were not down-regulated in 10 dpa fiber. Analyses of ESTs derived from cotton ovules supported an enrichment of transcription factors during early stages of fiber development [26]. Of the approximately 624 putative transcription factors represented on the microarray, 5 were regulated similarly to MYB109 and MYB2 and therefore were candidates to play a role in controlling fiber initiation (Contig3648, Contig65, Contig15274, WTOV_01-01-17R_C02 and TMIRS_117_D04.F). Semiquantitative rt-PCR confirmed that mRNA encoding Contig3648, which encoded a MYB-type transcription factor of unknown function, increased in 1 dpa fibers (Fig. 2). Contig15274 encoded a putative GLABRA2 transcription factor potentially in the same development pathway as MYB2 responsible for trichome development in Arabidopsis [25,38]. Another gene represented on the microarray (Contig2110) was also similar to GLABRA2 and was increased in expression in fiber initials, but was not statistically as well supported as Contig15274. A similar pattern of regulation of a GLABRA2-ortholog was also reported by Yang et al [26]. Contig17149, Contig16590 and Contig6466 also represent other regulatory genes potentially in the same developmental pathway as MYB2. Contig6466 encodes a putative TRANSPARENT TESTA GLABRA1 (TTG) gene and its up-regulation was statistically significant (probability = 0.03), but fell below the 2 fold cut-off set for identification of differentially expressed genes. Similarly, the down-regulation of a putative CPC gene (Contig16590) was below the 2 fold cut off but was statistically well supported (probability = 0.04). The other CPC gene (Contig17149) did not appear to be differentially regulated. A comparison of the translations of these putative CPC open reading frames with CPC from *Arabidopsis *suggests that the complete reading frame is represented (Fig. 4). Alignments of the putative CPC translations with the protein sequences of MYB2 from cotton, and GLI and CPC from *Arabidopsis *showed that the putative CPC that is down regulated in 1 dpa fiber has a single MYB domain (Fig. 4). The other putative cotton CPC was very similar to the CPC from Arabidopsis in the region of the MYB domain, though the NCBI conserved domain search did not identify it as a MYB domain. Both putative cotton CPCs apparently lacked a transcription activating domain just like CPC from Arabidopsis [24]. This was the first report of CPC-like sequences in cotton to our knowledge.

Genes other than transcription factors can have profound affects on expression of other genes. Expression of some other types of regulatory genes increased in 1 dpa fibers and persisted in 10 dpa fiber (additional file 1). Examples include receptor kinases, calmodulin, calmodulin binding proteins and lumen receptors (Contig15340, Contig16628, Contig2019, Contig17143 and Contig10804). RNA blot analysis confirmed differential expression of the calmodulin gene and a receptor kinase. (Fig. 2A). The calmodulin encoding mRNA expressed in the fiber samples was slightly larger than the most abundant calmodulin expressed in the whole ovule, indicating a unique calmodulin characterized by a different size was expressed in fibers. The receptor kinase differentially regulated as the fibers mature was detected in 10 dpa fiber. Apparently the level of the putative receptor kinase was not high enough in 1 dpa fiber to be detected. Semiquantitiative PCR confirmed differential expression of a lumen receptor and another receptor kinase (Fig. 2). Note that the semiquantitative PCR successfully detected the increase in expression between ovules and 1 dpa fiber but failed to detect the further increase in expression in 10 dpa fiber for either gene. It is likely that the method was not sensitive enough to detect a further increase.

### Transcription factor and other genes differentially expressed during fiber elongation

Genes potentially important in fiber elongation should be differentially expressed in 10 dpa fibers. Nine putative transcription factors were down-regulated in 10 dpa fibers compared to 1 dpa fibers (Contig13, Contig3089, Contig1984, Contig14677, Contig13751, Contig14961, Contig11028, WTOV_01-01-18R_G01, R10M_10R_E12_invR). This pattern of expression was confirmed by rt-PCR for a homeobox protein gene (Fig. 2). Nine putative transcription factors were up-regulated in 10 dpa fibers compared to 1 dpa fibers (Contig17592, Contig7886, Contig549, Contig4608, Contig18656, Contig17085, Contig11492, Contig15981, Contig1963). An mRNA encoding a potential calmodulin binding protein was also up-regulated in 10 dpa fibers consistent with the previously discussed increase in expression of a fiber specific calmodulin gene (Contig9400). An mRNA encoding Rho GDP dissociation inhibitor (GDPDI) was substantially increased between 1 dpa and 10 dpa fiber (TMIRS_147_F04.F). The GDPDI functions in a GTP mediated signaling pathway identified by GO analysis [32].

### Ca^+2 ^in fiber initials

**Figure 5:**
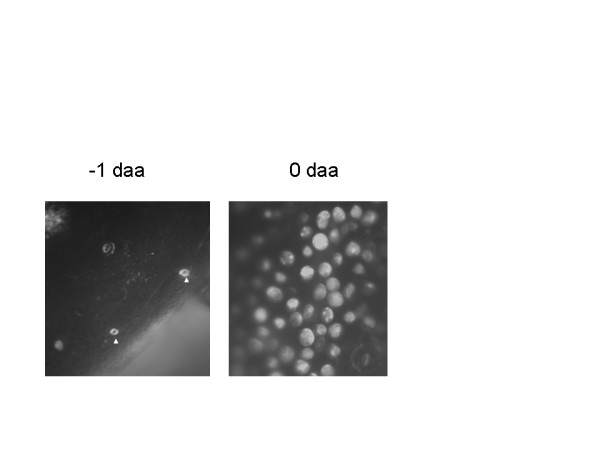
Ovules of the indicated age were stained with RhodFF. Water stained controls showed no auto fluorescence (data not shown). Arrow head indicate stained guard cells. The -1 dpa ovule is shown at 20× magnification to visualize a larger area. The 0 dpa ovule is shown at 40× magnification to allow easier visualization of stained cells.

The differential expression of calmodulin and calmodulin binding proteins indicated that calcium may play a role in fiber initiation. The distribution of Ca^+2 ^in the ovule epidermis was determined by staining ovules with RhodFF (Fig. 5). RhodFF had the same staining pattern observed for DiOC, indicating fiber initials had in increased demand for Ca^+2^. The only cells stained in -1 dpa ovules were the distinctive guard cells. Nascent fiber initials stained robustly in 0 dpa ovules. Staining was often "patchy" consistent with the dye not penetrating the sample well, though regions of higher Ca^+2 ^could not be ruled out. The vacuole did not appear to stain more than the cytosol indicating the Ca^+2 ^was not confined to the vacuole (Fig. 5). Abundant Ca^+2 ^in the ER was consistent with reports that this ion stabilizes ER membranes. The unequal distribution of Ca^+2 ^among ovule epidermal cells and the differential regulation of calmodulin and calmodulin binding proteins indicated that calmodulin mediated signaling could play an important role in fiber initiation and elongation.

## Discussion

The method developed to isolate RNA from fiber initials of *Gossypium hirsutum *allowed the direct isolation and analysis of genes expressed during fiber initiation. Profiling of gene expression on microarrays identified genes differentially regulated during fiber initiation and elongation. Statistical methods, inclusion of benchmark genes, RNA blot analysis and semiquantitative rt-PCR validated expression profiling data. Statistical analyses of GO also validated the expression profiling data by identifying an increase in protein biosynthesis in fiber initials and cell wall remodeling in elongating fibers consistent with previously reported aspects of fiber development [17,39].

Six of the genes used to validate the microarray experiments are MYB-type transcription factors. Transcription factors play a global role in control of gene expression. Additionally, their role in differentiation of leaf trichomes, a structure analogous to fiber, is particularly well characterized [12]. Expression of both MYB transcription factors (MYB2 and MYB109) important in fiber development were abundant in 1 dpa fibers and persisted into the elongation stage of fiber development. We have identified 5 transcription factors with a similar pattern of expression that could play a role in fiber development. Contig15274 was very similar to GL2 in *Arabidopsis *which acts down stream of GL1 to control trichome development [25,40]. These partial DNA sequences were about 50% identical to other GL2 type genes reported in cotton (AF530913 and AF530914) and 50% identical to an Arabidopsis GL2 gene (NM_106633). In *Arabidopsis *gl2 mutants result in expanded trichomes and proliferation of root hairs at position where root hairs would not normally develop [25]. An increase in expression of 2 genes similar to the TTG1 genes isolated from cotton which are able to restore trichome formation in the ttg1 *Arabidopsis *mutant was observed [38]. Two genes similar to CPC were observed in 1 dpa fiber. CPC acts as a negative regulator of trichome development in *Arabidopsis *[24]. One of the putative CPC genes was down regulated in 1 dpa fiber compared to ovules. The inhibitors described for *Arabidopsis *are not down regulated in trichomes; therefore it is not possible to draw a conclusion based in gene expression about which putative CPC gene in cotton was more likely involved in fiber development. If CPC genes in cotton act as inhibitors of fiber initiation, reducing expression of these genes with interfering RNAs would be expected to increase the number of fibers. Therefore a transgenic cotton line with reduced CPC expression could be agronomically valuable. In Arabidopsis GL1, GL2, TTG1, and CTC along with other regulatory genes control trichome development via lateral inhibition. The ability of cotton genes to complement the trichome mutant in *Arabidopsis thaliana *to restore trichomes supported by the presence of putative GL2 and CPC homologs in fiber clearly shows that fiber initiation and development of leaf trichomes use similar genetic mechanisms [12,38]. However analysis of the distributions of fiber initials stained with DiOC and previously published observations suggest that fibers often develop adjacent to each other (fig. 3A, 40× magnification) [4]. In the "lateral inhibition" model of trichome development trichomes do not normally develop in close proximity. Therefore other factors that interact with the "lateral inhibitory" pathway may be active in controlling fiber initiation.

GO analyses of genes differentially regulated during fiber development identified an increase in membranes specific to ovular trichomes. The hypothesis that membranes increased in fiber initials was confirmed by staining ovules with DiOC. DiOC may also stain mitochondria but the staining pattern was consistent with ER staining, though an increase in mitochondria in these metabolically active cells is also likely. The increase in ER was not evident until 0 dpa, was limited to fiber initials, was absent in a fiberless mutant, and was not observed in leaf trichomes. While the DiOC stain was not quantitative, the longer exposures required for leaves suggested ER levels were not high in leaf trichomes. We cannot eliminate the possibility that a transient ER increase occurred during leaf trichome initiation that was missed in these experiments. However, it is likely that the marked and long lasting increase in ER in fiber initials was unique to the ovular trichomes, indicating an early departure between the developmental programs that give rise to ovular and leaf trichomes. This increase in ER was consistent with the increase in golgi bodies reported in fiber initials. Abundant ER may play a role in biosynthesis and transport for components of the rapidly expanding cell membrane, cell wall and cuticle. Indeed, analysis of genes differentially regulated during fiber initiation and elongation identify numerous genes associated with these developmental pathways (see additional file 1). We propose that the increase in ER represented the first stages of fiber elongation since increase demands for cell membrane, primary cell wall, and cuticle production will persist through the elongation phase of fiber development.

DiOC stains fiber initials with sufficient contrast to allow direct counts of fiber initials. Digitalization of the DiOC stained image and counting DiOC stained ovule cells will allow us to develop protocols to identify cotton germplasms and mutations with increased density of fibers and follow the trait as it is crossed into elite germplasms.

A correlation between fiber initials, ER increase and Ca^+2 ^localization was also observed. ER membranes are stabilized by Ca^+2 ^therefore calcium is probably redistributed to fiber initials. Increased expression of a calmodulin gene unique to fibers, and differential expression of calmodulin binding proteins were also observed. It seems likely that a calmodulin mediated signaling pathway exists that either causes or responds to the redistribution of calcium into ER. Interestingly, deesterified pectins increase in fiber initials [41]. Deesterified pectins bind calcium; therefore it is likely that the cell walls may also compete for Ca^+2^. Manipulating expression of the calmodulin or manipulating calcium levels *in-vitro *should determine whether a calcium mediated pathway exists that causes or responds to the increase in ER and what role a calmodulin mediated response to Ca^+2 ^plays in fiber development.

GO analyses of genes up-regulated in fiber initials using UniProt cognates identified genes associated with a small GTPase mediated signal transduction pathway. This pathway has been implicated in transduction of signals in a variety of plant processes including response to light, pathogen responses and regulation of brassinosteroid biosynthesis [32,33]. This pathway may also play an important role with vesicle trafficking that is consistent with the increased level of Golgi bodies in fiber [4].

Genes that peak in expression during fiber initiation then decrease in expression during elongation would be expected to play a specific role in fiber initiation. Four well annotated genes with a fiber initiation-specific pattern of expression give potentially new insight into fiber initiation. A putative sterol sulfotransferase may alter brassinisteroids via sulfonation [37]. Brassinosterols play an important role in fiber initiation [2,21]. A prohibitin-like gene expressed in 1 dpa fiber may play a role in maintaining the fiber as a single cell [42]. The decrease in prohibitin mRNA correlated with an increase in the ploidy level of fibers cells [43]. A MATE efflux protein plays a role in root development and may play a similar role in fiber initiation [36]. Transducins play potential roles in signal transduction, have WD40 repeat motifs and may bind guanosine nucleotides [33,44,45]. This transducin was a fiber initiation specific component of GTP mediated signal transduction pathway different from the pathway identified by GO analyses. There are many other genes that are potentially differentially regulated available in the GEO submission (series accession number GSE6855). For example, 16 more transcripts were defined as fiber initial specific at 1.9 fold change of expression and 13 more transcripts were defined as fiber initial specific if a significance of 0.1 was used. Genes evaluated here may also become relevant as annotation of cotton genes improve. This information is available in GEO. Synchronously differentiating fibers represent a valuable developmental model to determine how developmental signals are integrated to control differentiation and elongation of fiber and how these signaling pathways differ between ovular and leaf trichomes.

## Conclusion

We present a new method of isolating RNA from very young fibers that allows the direct examination of genes expressed during fiber initiation. Sequencing cDNAs representing genes in a variety of cotton tissues, including fiber initials, has identified numerous genes not previously represented in GenBank. The expression profile of over 11,000 cotton genes, many unique to this investigation, was evaluated using microarrays. GO analysis identified an increase in genes associated with "membranes". Microscopic methods confirmed a marked increase in ER in fiber initials between 0 dpa and 1 dpa. Changes in expression of genes associated with Ca^+2 ^regulation were also observed and Ca^+2 ^concentrations were observed to be higher in fiber initials than surrounding cells. Genes potentially relevant to transcription regulation, brassinosterol regulation, cell cycle regulation and GTP mediated signal transduction were differentially regulated during fiber initiation. Genes associated with the "lateral inhibition" control of thrichome development in *Arabidopsis *were also present and many were differentially regulated during fiber development. A gene similar to CPC that acts as an inhibitor of trichome development in *Arabidopsis *was identified in fiber initials and appeared to possess the MYB domain but lack the transacting domain similar to its *Arabidopsis *counterpart.

## Methods

### RNA isolation, RNA blots and semiquantitative PCR

Stems, roots and the shoot (meristematic region) harvested from field grown DES119 cotton plants (2004) were frozen in liquid nitrogen and ground in liquid nitrogen in a Waring blender (Torrington, CT). Flowers from fields grown (2005) cotton plants (DES119 and ST4793R) were tagged with the date of anthesis and harvested 0 dpa, 1 dpa or 10 dpa. Fiber from 10 dpa ovules was dissected from the ovule, quickly frozen in liquid nitrogen and stored at -80°C. Polyribosomal RNA was isolated from 10 dpa fiber and 1 dpa ovules as described elsewhere [46-48]. Polyribosomal RNA was isolated from 1 dpa fiber by freezing freshly harvested 1 dpa ovules from 50 bolls in an excess of liquid nitrogen, adding about 0.1 g glass beads (Sigma, Atlanta, GA) and vortexing for 5 min. After the liquid nitrogen evaporated but before the sample warmed, 20 ml of the first buffer for polyribosomal RNA isolation was added and the intact ovules removed by filtering through cheese cloth. Free-polyribosomal RNA, membrane bound-polyribosomal RNA and total polyribosomal RNA was isolated as usual. Between 25 μg and 65 μg of total polyribosomal RNA was typically recovered. RNA quality was confirmed on a BioAnalyzer (Agilent, Palo Alto, CA).

RNA was separated on a 1.2% agarose gel (Phosphate buffer, pH6.5) and transferred to positively charged Nytran membrane (Roche, Alameda, CA) as described elsewhere [49]. The probe was amplified from the 3' end of the selected transcripts using the PCR DIG synthesis Kit (Roche). The blot was hybridized, rinsed and visualized following the instructions in the DIG Wash and Block Buffer Set (Roche). RT-PCR and Semiquantitative PCR were described in Taliercio and Kloth [50]. Primer sequences are presented in Table 1.

### EST assembly and analysis

ESTs libraries representing unnormalized sequences were prepared as described in Taliercio et al. [51] and a comprehensive list of tissues represented is shown in Table 2. Vertis Biotechnologie (Freising-Weihenstephan, Germany) made the normalized libraries. Normalization brings the frequencies of most mRNAs within a narrow range [52]. Normalized cDNA libraries representing oligodT primed or randomly primed RNA were made by pooling RNA from meristematic regions, 1–3 dpa fibers, 7 week old roots, 3–10 week old stems. Complementary DNA representing the pooled RNA was PCR amplified 16 (oligodT) or 21(random) cycles using sequences included in the primers and normalized 1 time over an hydroxylapatite column. The single stranded fraction was amplified 11 cycles and cDNAs >~0.6 kb were cloned into the BamHI-EcoRI site of pBSIIsk+. Aliquots of the cloned cDNA were electroporated into TOP10 cells (Invitrogen) and sequenced from the 3'end at the MidSouth USDA/ARS genomics facility. Selected clones were also sequenced from the 5'end. Sequences were carefully trimmed to remove vector sequences and deposited in GenBank Table 2.

These ESTs were assembled into contigs with *Gossypium hirsutum *ESTs from GenBank using CAP3 [53]. ESTs that do not assemble with others are called singletons. Orientation of the assemblies were determined by the known orientation of any of the component ESTs, presence of a polyA tail at the end of the contig, and/or a strong match (<E-24) with a protein sequence. An ACCESS database archiving the assembly was used to analyze the distribution of ESTs. We reasoned that contigs consisting of ESTs from the new libraries would be good sources of genes expressed in fiber initials (also stems, shoots and roots) since fiber initials were not previously well represented in GenBank. Probes spotted on the microarray were 40 nt-60 nt oligonucleotides identified using "picky (version 2.00)" software that represented the contigs and singletons unique to the new libraries but not contigs and singletons assembled from ESTs already in GenBank [54]. A microarray using these probes and including probes representing seventeen genes with well characterized expression in young fiber already in GenBank was made by Agilent (Palo Alto, CA). Information about the microarray has been deposited in the Gene Expression Omnibus (GEO) at NCBI under the accession number GPL4739. The complete sequences of the genes represented on the microarray were included on the platform submission to GEO.

The Contigs and singleton sequences used on the microarray were used to query GenBank's nonredundant database and the TAIR database with BLASTx. The multiple matches from GenBank were saved, but only the best match that was better than E^-10 ^were saved from the TAIR database. Approximately 74% of the assembled sequences had TAIR cognates that met the query criterion. A similar method was used to identify UniProt cognates. The complete gene sequences for all of the genes represented on the microarray, the sequences of the probes printed on the microarray, expression profiles of all of the genes on the microarray and more extensive BLAST annotation are available as GEO series accession number GSE6855.

### Hybridization and analysis of microarrays

RNA isolated from 3 biological replications of 1 dpa intact ovules, 1 dpa fiber, or 10 dpa fiber was labeled with CY3 or CY5 using the MicroMax kit (Perkin Elmer, Wellesley MA). Biological replications were harvested on different days. Three microarrays were hybridized with labeled cDNA presenting 1 dpa fiber and 1 dpa ovules including one dye swap. Three microarrays were hybridized with cDNA representing 1 dpa fiber and 10 dpa fibers including one dye swap. All hybridizations, array scanning and basic analyses were done by MoGene (St Louis, MO). To test for treatment differences, analysis of variance (ANOVA) was performed using SAS (Cary, NC). The F-test was used to test for significance of expression levels. Since hybridization with 1 dpa fiber was the common treatment, an ANOVA using all treatment and assuming common variance among the 3 treatments resulted in a better estimate of error and an increase the precision of the study. The experimental design including all 6 microarrays was an Incomplete Block design with 3 treatments taken 2 at a time. Some of the genes had more than 1 probe on a plate and some genes had more than 1 spot for a single probe resulting in several levels of subsamples. Therefore, the potential sources of variability (random effects) for each gene represented on the array are: 1) overall block effect of chip, 2) chipXtreatment, 3) oligo within chip and treatment and, 4) residual (spot to spot variability for a given probe). A detailed analysis of the statistics will be published in the Proceeding of Applied Statistics in Agriculture. Unless otherwise noted, 2 fold differences in expression supported by a probability of 0.05 or less are considered significant and probabilities of 0.01 or less are considered highly significant. We note that the statistical analysis of three of the benchmark genes fell slightly outside of this range. Data from this microarray series has been deposited in GEO under accession number GSE6855.

### Staining ovules and leaves with DiOC and RhodFF

Cotton plants (ST4793R) were grown in the greenhouse (winter 2005–2006 and spring 2006)) supplemented with 6 h of halogen light. Small unexpanded leaves were harvested from ~3 week old plants, washed in water and incubated for 30 min in 25 μM 3, 3'-Dihexyloxacarbocyanin iodide (DiOC) (Sigma), or in water. Ovules harvested from bolls -1 dpa, 0 dpa, 1 dpa or 3 dpa were washed briefly in phosphate buffered saline (PBS) and incubated for 30 min in 25 μM DiOC or in water. Ovules were rinsed in PBS and also stained in 10 uM RhodFF (Invitrogen), washed with 1 ml PBS and incubated at room temperature for 30 min before visualization. Midribs were cut from the leaves and the leaves were places on a slide and covered with 50% glycerol and a cover slip. The ovules were cut in quarters, with one cut along the chalazal ridge to help orient the section and covered in 50% glycerol and a cover slip. Stained ovules and leaves were visualized using fluorescence on the Axioskop 2 using the FITC/EGFP filter (41012) for DiOC or the red filter (41035) for RhodFF (Chroma, Rockingham, VT). Images are of the central portion of the ovule unless otherwise noted. Images were taken with the Axiovision (Zeiss, Thornwood, NY) camera using the Axiovision 4.4 software (Zeiss). Exposures were in the range of 50 ms for stained ovules and much longer for unstained ovules and stained or unstained leaves. DiOC stained images were digitalized and stained cells counted using the nonproprietary software Image J.

## Authors' contributions

Deborah Boykin was instrumental in designing the microarray, wrote the SAS program to do the statistical analysis of the microarray data and formatted the data for this manuscript. Earl Taliercio designed the experiments, prepared the unnormalized libraries, archived and annotated the sequences and evaluated the diverse varieties of data presented in this manuscript. He also prepared this manuscript. The authors read and approved the final manuscript.

## Supplementary Material

Additional File 1 Gene expression in fiber initials. These data show expression and tentative identification of the sequences represented on the microarray. Click here for file
